# Challenging diagnosis of leprosy in a psychotic homeless patient with atypical clinical manifestations: an interesting case report

**DOI:** 10.1186/s12879-021-06242-0

**Published:** 2021-06-07

**Authors:** Hendra Gunawan, Reyshiani Johan, Pati Aji Achdiat, Oki Suwarsa

**Affiliations:** Department of Dermatology and Venereology, Faculty of Medicine, Universitas Padjadjaran – Dr. Hasan Sadikin General Hospital, Jl. Pasteur No. 38, Bandung, 40161 Indonesia

**Keywords:** Atypical manifestation, Case report, Histopathological examination, Psychotic homeless leprosy

## Abstract

**Background:**

A decision to diagnose certain skin diseases in patient undergoing psychotic break is challenging; this includes establishing the diagnosis of leprosy. Diagnosis of leprosy is established if there is at least one of the three cardinal signs of leprosy. Histopathological examination is not a gold standard, but remains useful in atypical or clinically suspicious cases.

**Case presentation:**

We report for the first time, an interesting case of leprosy with atypical clinical manifestations in a psychotic homeless male with unknown history of present illness. Upon examination, hypopigmented macules, hyperpigmented macules, and plaques were observed, with unclear sensation impairment. Peripheral nerve thickening and acid-fast bacilli from slit-skin smear were not found. Histopathological examination from hypopigmented macule on the upper right limb showed no granulomatous reaction and other histopathological features of leprosy. Although the condition did not fulfill the cardinal signs of leprosy, we found lagophthalmos, claw hands, pseudomutilation of fingers and toes. Therefore, the diagnosis of suspected leprosy was established. The patient was hospitalized and attempts to administer oral rifampicin and clofazimine were made. Several days after treatment, annular erythematous macules appeared on the patient’s face, abdomen, and back. Histopathological examination results on sample taken from erythematous macule and right sural nerve were consistent with the diagnosis of leprosy with reversal reaction.

**Conclusion:**

In certain conditions, histopathological examination of the skin and nerves are a highly rewarding test in establishing a diagnosis of leprosy.

## Background

Leprosy is a chronic granulomatous infection caused by *Mycobacterium leprae* (*M. leprae*) and *Mycobacterium lepromatosis* [1–3], affecting particularly peripheral nerves and skin.^2^ Leprosy remains a public health problem, especially in developing countries. It can cause permanent damage to the eyes, hands, and feet, and can be physically, psychologically, and socially devastating if not diagnosed and treated early [4].

Diagnosis of leprosy is established if there is at least one of the three cardinal leprosy signs such as hypopigmented or erythematous macule with loss of sensation, thickening of the peripheral nerves with impaired function, and acid-fast bacilli (AFB) found on slit-skin smear examination. Histopathological examination is one among many examinations that can help establish the diagnosis of leprosy, especially for cases with atypical clinical manifestations. This examination also serves to rule out differential diagnoses, determine therapy and prognosis, and useful for research purposes [5, 6]. The aim of this case report was to show how to establish the diagnosis of leprosy in psychotic homeless patients with atypical manifestations.

## Case presentation

**Fig. 1:**
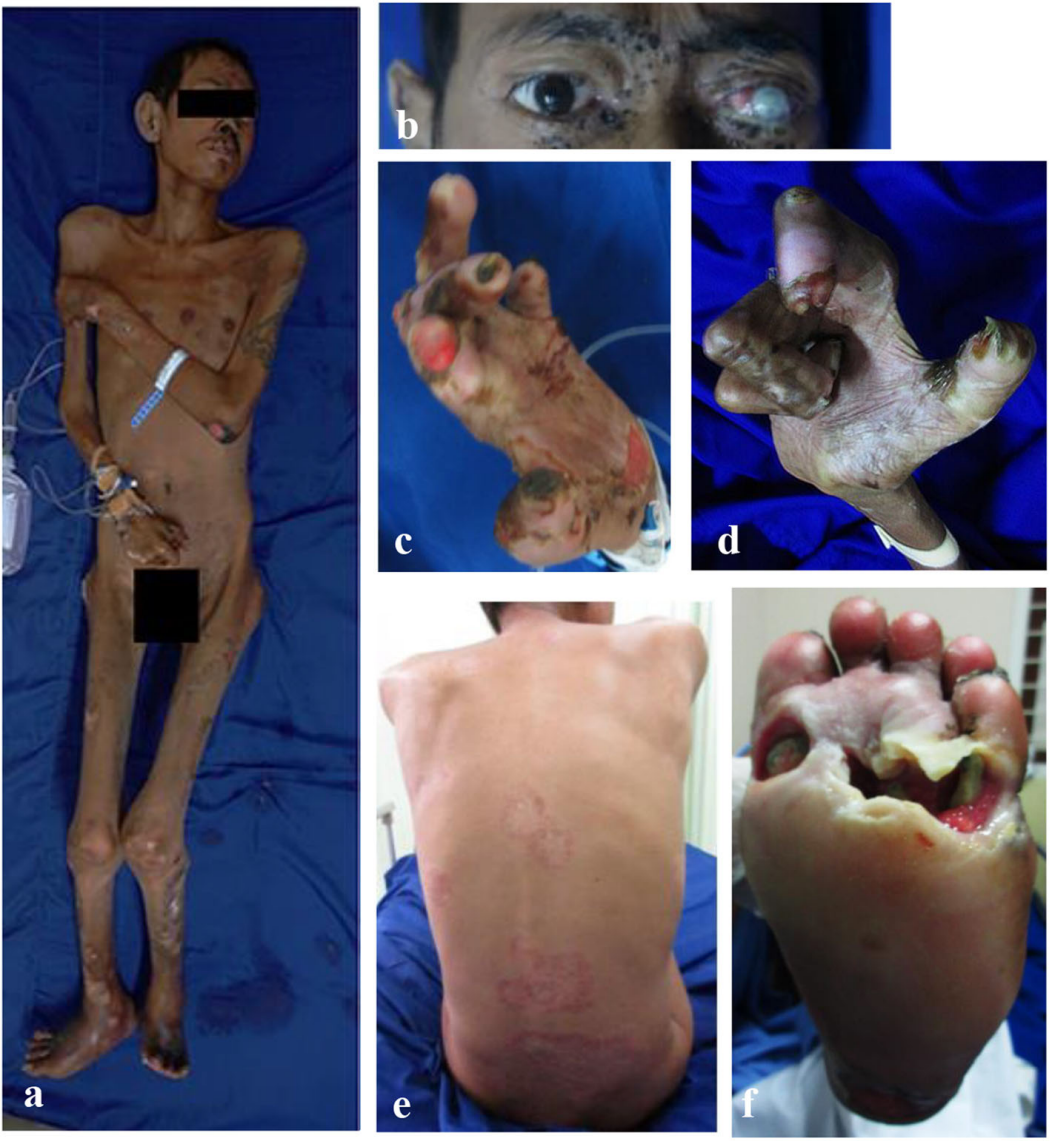
Clinical manifestations of leprosy. (**a**) Hypopigmented macules, hyperpigmented macules and plaques (**b**) Lagophthalmos on the left eye and opacity of left cornea, (**c**-**d**) Claw hands and pseudomutilation, (**e**) Annular erythematous macules on the back, (**f**) Multiple ulcers on the foot

A male, unknown age, homeless with unknown history of present illness was taken by a social service officer to the Emergency Unit after the patient was found lying on the street in a state of illness and unattended. The patient does not want to be spoken to, sometimes making “harsh” words in a tantrum. The patient presented with hypopigmented macules, hyperpigmented macules and plaques with unclear sensation impairment (Fig. 1a), multiple erosions and ulcers on both hands, buttocks, and feet (Fig. 1f). There were no thickening of peripheral nerves and slit-skin smear (both ear lobes, both cheeks, forehead, chin, both buttocks and additional three hypopigmented macules) were negative. Otherwise, we found lagophthalmos of the left eye (Fig. 1b), defect on the left nostril, claw hands, pseudomutilation of fingers and toes, and contracture of hands (Fig. 1c-d). Histopathological examination of samples taken from hypopigmented macule on the upper right limb (1st biopsy) revealed that the epidermis was within normal limits, with mild lymphocytes infiltration at the perivascular and surrounding glands. There were no granulomatous reactions and other histopathology features of leprosy. Laboratory examination revealed that the patient had anemia, hypoalbuminemia, electrolyte imbalance, and anti-Phenolic Glycolipid (PGL)-1 Immunoglobulin (Ig) M and IgG titers were 237 μ/ml and 76 μ/ml (cut off 605 μ/ml), respectively. Radiography showed muscle contractures in the proximal to distal phalanges of both hands and multiple malunion fractures of bilateral metatarsal and phalanges of both feet accompanied by gangrene and osteomyelitis. The criteria of leprosy for this patient were not fulfilled, therefore we diagnosed him as suspected leprosy. The patient was hospitalized, and attempts to administer oral rifampicin 600 mg (every month), clofazimine 300 mg (every month) and 50 mg (each day) were made. He was bathed with soap (two times daily), and treated with using 0.9% sodium chloride wet dressing (two times a day for his ulcers and crust), and 2% mupirocin cream (two times a day). The patient was being treated using multidisciplinary approach, in collaboration with seven other departments. The Internal Medicine Department administered packed red blood cells and albumin transfusion, intravenous antibiotic, and electrolyte correction to improve the patient’s general condition. The Plastic Surgery Department did wound toilet for ulcer with hydrogel. The patient was planned for debridement and negative pressure wound therapy (NPWT) by the Orthopedic and Traumatology Department. The patient received three meals per day with additional administration of liquid food to improve his malnutrition from the Nutritional Department. The patient was diagnosed with unclassified psychosis with differential diagnosis of other mental disorders due to damage and dysfunction of the brain and physical disease by the Psychiatric Department, with no need for medications. The Ophthalmology Department gave multiple eye drops containing antibiotics (tobramycin, ofloxacin, chloramphenicol, and polymyxin B sulfate) for the left eye. The patient got flexibility exercise of extremities from Physical Medicine and Rehabilitation Department.

**Fig. 2:**
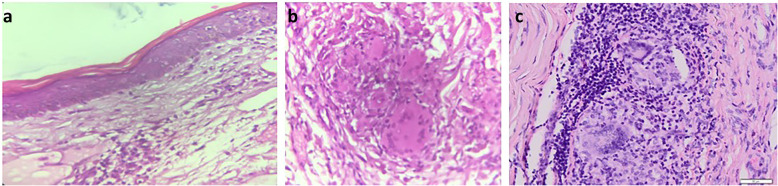
Histopathological examination of the skin and nerves. (**a**) Histopathological examination of sample taken from the annular erythematous macule found on the back revealed extracellular edema in the superficial dermis (hematoxylin and eosin, × 40 magnification) and (**b**) granulomatous reaction composed of epithelioid cells and multinucleated giant cells at perivascular, perineural, and surrounding eccrine glands (hematoxylin and eosin, × 100 magnification). (**c**) Histopathological examination of the right sural nerve revealed granuloma reactions and multinucleated giant cells (hematoxylin and eosin, × 100 magnification)

On the 36th day of observation, there were erythematous macules on the face, abdomen, and back (Fig. 1e) that we diagnosed as a severe reversal reaction. There were no thickening of peripheral nerves and slit-skin smear (both ear lobes, both cheeks, forehead, chin, both buttocks and additional three erythematous macules) were negative. We did punch biopsy (2nd biopsy) on samples taken from the erythematous macules on the back and incision biopsy on samples from the right sural nerve. The Orthopedic and Traumatology Department did debridement of the feet with general anesthesia simultaneously. The patient received 40 mg oral prednisolon every day for 2 weeks and tapered off every 2 weeks. From histopathological examination of the right sural nerve, granulomatous reactions were found with proliferation of epithelioid cells, lymphocytic inflammatory cells, and multinucleated giant cells, which supported the diagnosis of leprosy (Fig. 2c). From histopathological features of the skin, epidermal atrophy was found with multinucleated giant cells at perivascular, and epithelioid cells at perivascular, perineural, and surrounding eccrine glands, which supported the diagnosis of borderline tuberculoid (BT) leprosy (Fig. 2a-b). The patient’s final diagnosis was BT leprosy with severe reversal reaction and secondary disabilities of the eye, hand, and feet with osteomyelitis, unclassified psychosis, and malnutrition. Treatment for leprosy will be continued until 6 months with tapering of corticosteroid for reversal reaction.

## Discussion and conclusion

Diagnosis of leprosy is established upon finding at least one of the three cardinal signs of leprosy, as have been established by the World Health Organization (WHO). These include hypopigmented or erythematous skin lesions with loss of sensation, thickening of the peripheral nerves with impaired function, and AFB found on slit-skin smear examination [1, 7]. These cardinal signs were not found in this patient, including difficulty to assess the sensation impairments of skin lesions due to the patient suffering from psychosis.

Histopathological examination of the skin and nerves as well as serological examination are investigations that can be done to help diagnose leprosy [5, 8]. Although not a gold standard for diagnosis [7], histopathological examination can help establish leprosy cases that are not typical or still clinically suspected [5, 7, 9]. In tuberculoid leprosy, commonly found histopathological features of the skin include infiltration of granulomas around blood vessels, nerve vessels, and musculus arrector pili, with a small number of lymphocytes. Multinucleated giant cells are found [10, 11] and *M. leprae* can be found in small amounts or not found at all [10–12]. In the current case, the histopathological features of samples taken on first biopsy of the hypopigmented macules found on the upper limb showed no epidermal changes, with mild infiltration of lymphocytes at the perivascular and surrounding glands, without granulomatous reaction and other histopathological features of leprosy. However, the patient was diagnosed as suspected leprosy due to the lagophthalmos, claw hands, pseudomutilation, and contracture of the extremities. After 1 month of leprosy treatment, erythematous macules appeared on the face, abdomen, and back. These macules were asymmetric and arranged in annular configuration, sharply marginated, and scaly, as were most often seen in patients with BT leprosy. Therefore, the patient was diagnosed as BT leprosy with a severe reversal reaction.

In leprosy, before the reaction is clinically apparent, histopathological features often show diffuse extracellular edema in the superficial dermis. However, when the reaction is clinically apparent, the edema and proliferation of fibroblasts may be profuse. In reversal reactions, the granuloma becomes completely composed of epithelioid cells and giant cells. An important feature of a severe reaction is the breakdown of the granuloma or even liquefaction necrosis [13]. In this patient, the histopathological features from the second biopsy on the annular erythematous macule found on the back showed extracellular edema in the superficial dermis with granulomatous reaction composed of epithelioid cells and multinucleated giant cells at perivascular, perineural, and surrounding eccrine glands. Based on these histopathological features, the diagnosis of BT leprosy with reversal reaction was established.

The histopathological features of nerve biopsy are divided into five groups [14]. In lepromatous leprosy (LL), bacteria are found in large amount and some are arranged in a globus, spread on each layer of neurons, macrophages, and cytoplasm in vascular endothelial cells [6, 15], while in borderline lepromatous (BL) leprosy, bacteria are found to be abundant, with changes in the structure of the perineurium and endoneurium. In mid-borderline (BB) leprosy, large numbers of bacteria are found, accompanied by epithelioid cells, small amounts of lymphocytes, and abnormal perineurium structures. Granuloma cell infiltrates with a small number of bacteria were found in BT leprosy [15], while granulomas containing histiocytes and multinucleated giant cells without the existence of bacteria were found in tuberculoid leprosy (TT) [6]. In this case, the histopathological examination on samples taken from the sural nerve showed granulomatous reactions with the proliferation of epithelioid cells, lymphocytic inflammatory cells, and multinucleated giant cells, consistent with the diagnosis of tuberculoid leprosy.

A study conducted by Ashok Kumar et al. (1995) in India reported that the histopathological examination of the nerves can establish the diagnosis of leprosy in 26 of 27 patients [16]. An Ethiopian study by Nilsen et al. in 1985 demonstrated that 64% of the 81 patients enrolled in the study were diagnosed with leprosy through histopathological examination of the nerves [17]. The other report showed that histopathological examination of the nerves can be used to diagnose leprosy in patients without skin lesions [18].

A delay in establishing diagnosis and providing treatment for leprosy patients can result in disability, both primary and secondary [19, 20] which can affect the face, hands, feet, and other body parts [21]. Primary disability is caused by direct infiltration of *M. leprae* in Schwann cells or direct damage due to leprosy reaction [22, 23]. Secondary disability occurs due to untreated disability and undergoes progression [24] which causes loss of sensory and motor function [22], can be in the form of wounds, ulcers, and contractures in the joints [19, 20]. In this patient, we observed secondary disabilities of the eyes, hands, and feet.

Establishing the diagnosis of certain skin diseases in a patient undergoing psychotic break is challenging; this includes leprosy. In establishing the diagnosis of leprosy in psychotic patients, understanding the patients’ skin sensation is important. For instance, determination of sensory impairment in a psychotic patient may be quite difficult. Moreover, in an atypical clinical manifestation of leprosy, the demonstration of classical histological features of leprosy or AFB in cutaneous lesions is difficult, hence a reasonable period of observation is recommended if the diagnosis cannot be confirmed at the time of presentation. In the current case, there were skin lesions with unclear sensation impairment, without peripheral nerves thickening, and without a finding of AFB. In addition, the first histopathological examination of skin lesion samples showed neither granulomatous reaction nor other histopathology features of leprosy. After attempts to treat the patient with antileprosy were made, annular erythematous macules appeared and histopathological features of skin lesion and sural nerve supported the diagnosis of leprosy with reversal reaction.

Multidrug treatment (MDT) of paucibacillary leprosy consists of rifampicin and dapsone for 6 months [1, 25]. Due to the patient’s anemia, dapsone was replaced with clofazimine. Systemic corticosteroid is the most common drug used for the treatment of leprosy reaction, due to its anti-inflammatory and immunosuppressive effect [1, 13]. In accordance with WHO guidelines, the corticosteroid dose for early treatment is started at 40 mg/day (prednisolone 40–60 mg/day), then gradually tapered off by 5–10 mg every 2 weeks, in a standard 12-week treatment course [26]. In this patient, reversal reaction improvement occurred in the 14th day of observation, with a resolution of skin lesions. The patient was also treated with a multidisciplinary approach due to psychiatric illness, systemic abnormalities, and leprosy-related disabilities.

This case report highlights an interesting case of leprosy in psychotic homeless patient with atypical clinical manifestations. In certain condition, histopathological examination of the skin and nerve are a highly rewarding test in establishing the diagnosis of leprosy.

## Data Availability

Not applicable.
